# Muscle Activation in Middle-Distance Athletes with Compression Stockings

**DOI:** 10.3390/s20051268

**Published:** 2020-02-26

**Authors:** Diego Moreno-Pérez, Pedro J. Marín, Álvaro López-Samanes, Roberto Cejuela-Anta, Jonathan Esteve-Lanao

**Affiliations:** 1 Departament of Education, Research and Evaluation Methods, Comillas Pontifical University, 28015 Madrid, Spain; dmperez@comillas.edu; 2 CYMO Research Institute, 47140 Valladolid, Spain; pjmarin@checkyourmotion.com; 3 School of Physiotherapy, Faculty of Health Sciences, Universidad Francisco de Vitoria, 28223 Madrid, Spain; 4 Department of Physical Education and Sports, University of Alicante, 03690 Alicante, Spain; roberto.cejuela@ua.es; 5 All in Your Mind Training System, Mérida 97134, Mexico; jonathan.esteve@allinyourmind.es

**Keywords:** surface electromyography, compression garment, gradual-elastic compression stockings, muscular fatigue, endurance athletes

## Abstract

The aim of this study was to evaluate changes in electromyographic activity with the use of gradual compression stockings (GCSs) on middle-distance endurance athletes’ performance, based on surface electromyography measurement techniques. Sixteen well-trained athletes were recruited (mean ± SD: age 33.4 ± 6.3 years, VO_2max_ 63.7 ± 6.3 mL·kg^−1^·min^−1^, maximal aerobic speed 19.7 ± 1.5 km·h). The athletes were divided into two groups and were assigned in a randomized order to their respective groups according to their experience with the use of GCSs. Initially, a maximum oxygen consumption (VO_2max_) test was performed to standardize the athletes’ running speeds for subsequent tests. Afterward, electromyographic activity, metabolic, and performance variables for each group were measured with surface electromyography. In addition, blood lactate concentration was measured, both with and without GCSs, during 10 min at 3% above VT2 (second ventilatory threshold), all of which were performed on the track. Next, surface electromyography activity was measured during a 1 km run at maximum speed. No significant changes were found in electromyography activity, metabolic and performance variables with GCSs use (*p* > 0.164) in any of the variables measured. Overall, there were no performance benefits when using compression garments against a control condition.

## 1. Introduction

Graduated compression stockings (GCSs) are socks that create a compressive pressure around the muscle, bone, and connective tissue, with this pressure higher in the ankle area and gradually decreasing until the knee [1]. In addition, compression garments were originally used to treat deep vein thrombosis [2] and venous insufficiencies [1,3]. Thus, several studies have demonstrated an increase in the venous velocity, a reduction of venous pooling, and improvement in venous return in hospital patients who wore GCSs [1,4]. Although there are no significant changes in heart rate associated with the use of GCSs in endurance events, [5,6,7,8] the interest in the sports sciences field in GCS application and commercialization is highly increasing [9]. 

A decrease in the concentration of metabolites associated with compression garment use may have benefits during submaximal running efforts [10]. Berry and McMurray [11] hypothesized that a reduced blood lactate concentration associated with the use of compression stockings could be due to greater blood flow removal during exercise with CGSs. In addition, it has been described that GCSs lead to improvements in blood lactate concentration clearance during continuous sports, such as cycling [12]. However, other studies reported no changes in blood lactate concentration with the use of GCSs in endurance efforts [6,7,8,13]. The differences between studies could be attributed to the different methodologies used [6,7]. Thus, the benefits of using GCSs while running are not entirely clear at the metabolic and cardiovascular levels. Moreover, the possible improvements in muscle recruitment with the use of GCSs during dynamic actions are unknown.

One method to measure the fiber recruitment is electromyography (EMG). This type of measurement technique comprises the sum of the electrical contributions made by the active motor units (MUs), that are detected by electrodes placed on the skin overlying the muscle. The information extracted from the surface EMG is often considered a global measure of MU activity because of the inability of the traditional (two electrode) recording configuration to detect activity at the level of single MUs, which allows the measurement of the electrical signal during a muscular action [14]. Raez et al. [15] defined EMG as the acquisition, recording, and analysis of electrical activity produced by nerves and muscles through electrode surface electromyography, which is a noninvasive method that allows the evaluation of muscle recruitment during dynamic efforts [16]. Most recent research on compression garments by means of EMG has mainly been focused on the relationship between intramuscular pressure and EMG responses during concentric isokinetic muscle contractions [17]. Likewise, previous research underlined the relationship between the use of compression garments and the perception of lower muscle pain [5,18], greater comfort, and a lower subjective perception of effort (RPE; rating of perceived exertion) [18]. According to Varela-Sanz [13] there seems to be a tendency to run faster with a lower perception of effort. If there are no clear metabolic or cardiovascular benefits, the benefits may be found in a change in muscle recruitment between the thigh muscle and the leg (i.e., triceps surae).

The aim of this study is to evaluate changes in electromyographic activity with the use of the use of gradual compression stockings (GCSs) on middle-distance endurance athletes’ performance, based on surface electromyography measurement techniques. We hypothesize an improvement in submaximal (i.e., 10 min at 3% above VT2 (second ventilatory threshold)) and maximal conditions (i.e., 1 km at full speed) with the use of gradual compression stockings compared to control condition.

## 2. Materials and Methods

### 2.1. Participants

Fourteen male and two female athletes reported to the laboratory three times with 72 h hours between protocols (mean ± SD, age 33.2 ± 7.2 years, VO_2max_ 63.7 ± 6.3 mL·kg^−1^·min^−1^, maximal aerobic speed 19.7 ± 1.5 km·h^−1^, 4 min and 18 s at 1500 mL). All athletes had competed in the Spanish Track and Field Championships, and some of them had won medals at the National Track Veterans’ Championships.

Before the beginning of the study, all subjects gave written informed consent in accordance with the Declaration of Helsinki [19]. The protocol was approved by the Ethics Committee of the University. The athletes were randomly assigned to either an experimental group, with GCSs (EXP), or control group without CGSs (CNT). There were no significant changes in the descriptive variables between groups (*p* < 0.050).

### 2.2. Experimental Design

Day 1: A maximum oxygen consumption (VO_2max_) test was performed in order to define the subjects’ running speeds for consecutive tests. After a standardized warm-up of 20 min of continuous running on a treadmill (Technogym Run Race 1400 HC, Gambettola, Italy) at 60% of their maximum heart rate and a block of dynamic warm-up [20], subjects performed a VO_2max_ test with a gas analyzer (VO2000, Medical Graphics Corporation, St. Paul, MN, USA). The variables that were measured were oxygen uptake (VO_2_), pulmonary ventilation (VE), ventilatory equivalents for oxygen (VE·VO_2_^−1^) and carbon dioxide (VE·CO2 VE·VO2^−1^), and end-tidal partial pressure of oxygen (PETO2) and carbon dioxide (PETCO2). VO_2max_ was recorded as the highest VO_2_ value obtained for any continuous 30 s period during the test. The VT1 was determined using the criteria of an increase in both VE·VO2^−1^ and PETO2 with no increase in VE·VCO2^−1^, whereas the VT2 was determined using the criteria of an increase in both VE·VO2^−1^and VE·VCO2^−1^ and a decrease in PETCO2 [21]. Two independent observers detected VT1 and VT2. If there was disagreement, a third investigator was consulted. The maximal aerobic speed was associated with the last completed 30 s stage before the exhaustion, which was associated with VO_2max_ [21]. The protocol started with a gradient of 1% at a speed of 10 km·h^−1^, with increments of 0.3 km·h^−1^ every 30 s until the maximum exhaustion [21]. The tests were performed in the Exercise Physiology Laboratory of the Universidad Europea de Madrid (i.e., 600 m altitude). All evaluations were performed at the same time of day (i.e., evening, between 7:00 p.m. and 9:00 p.m.) and under similar environmental conditions (i.e., 20–22 °C temperature, 60–65% relative humidity) to avoid effects associated with circadian rhythms on performance [22]. 

Days 2 and 3: Each group had to perform the same training session with compression garments (EXP) and without GCSs (CNT), with a recovery period of 72 h between the two sessions. One group was assigned to use GCSs only on the first day, and the other group was assigned to use GCSs only on the second day (the athletes served as their own controls). On the day that GCSs were not used, the athletes used traditional socks. The participants wore GCSs (Medilast Sport, Lleida, Spain) with degressive pressure (15–20 mm Hg at the ankle; 88% Polyamid, 12% Elasthane) from the ankle to the calf area (always under the supervision of a member of the investigators’ team.). The compression was similar to that used in the medical field [23]. 

### 2.3. Surface Electromyographic Activity (EMG) 

EMG was measured according to the electrical activity (EA) recorded with a telemetric system (BTS Pocket EMG, Garbagnate M.se, Italy). The information extracted from the surface EMG give global and, rarely, individual indications of motor units activity [24]. A sampling frequency of 1 kHz was used. Preamplifiers placed next to the measuring electrode allowed ruling out the influence of likely movements of the wires on the measurement. Signals from the EMG were band-pass filtered (10–400 Hz), and the root mean square (RMS) was analyzed. Bipolar surface EMG electrodes (Al/AgCl, discs of 10 mm diameter) with an inter-electrode distance of 24 mm were placed on the bellies of the vastus lateralis (VAL), vastus medialis (VAM), rectus femoris (RF), biceps femoris (BF), gastrocnemius (GAM), and soleus (SOL) in accordance with the Surface EMG for Non-invasive Assessment of Muscles [25]. 

We evaluated EMG during two footraces: (a) 10 min at 3% above VT2 (*t* > VT2) (i.e., represents a submaximal effort), (b) 1 km at full speed (t1km) (i.e., represents a maximal effort). These runs were performed on the athletics track with a 3 min break in between. All evaluations were performed at the same time of day (i.e., evening, between 7:00 p.m. and 9:00 p.m.) and under similar environmental conditions (i.e., 22–24 °C temperature, 55% relative humidity).

### 2.4. Metabolic, Perceptual and Performance Variables 

The concentration of blood lactate concentration (mmol/L^−1^) was measured at *t* > VT2 with a blood lactate analyzer (Lactate Pro Arkray INIC, Amstelveen, NED). The subjective perception of effort (RPE) was measured using the Borg scale [26]. For the performance variable, a stopwatch was used to measure the time (min) subjects took to run t1km. 

### 2.5. Statistical Analysis

The data set obtained was analyzed with the SPSS Statistics 19 software (SPSS Inc., Chicago, IL, USA). *T*-tests were applied to related samples, both to verify that there were no differences in matching the subjects and to observe the differences in the sports performance variables. All data were expressed as mean (M) and standard deviation (SD). Homogeneity of variance was tested with the use of a Kolmogorov–Smirnov test and Lilliefors correction. The level of statistical significance was set at *p* < 0.05. The significance level was set at 0.05. Cohen’s formula for effect size (ES) was used and the results were based on the following criteria: trivial (0–0.19), small (0.20–0.49), medium (0.50–0.79), and large (0.80 and greater) [27].

## 3. Results

### 3.1. Metabolic and Perceptual Variables at Submaximal Efforts (t > VT2)

According to the metabolic demands and perceptual variables, no statistical differences were founded in the different conditions measured in the study between GCSs and CNT conditions, such as heart rate (182.6 ± 10.1 versus 182.6 ± 10.0, *p* = 1.000, ES < 0.01, trivial), blood lactate concentration (mmol·L^−1^) (8.3 ± 2.1 versus 7.9 ± 2.4, *p* = 0.476, ES = 0.20, small), and RPE (8.5 ± 1.0 versus 9.0 ± 0.6, *p* = 0.301, ES = 0.33, small). 

### 3.2. Perceptual and Performance Variables at Maximal Effort (t1km)

Perceptual and performance variables did not reach statistical significance during t1km—GCS versus CNT: RPE (9.9 ± 0.3 versus 10.0 ± 0.0; *p* = 0.164; ES = 0.39, small), speed (19.2 ± 1.7 versus 19.1 ± 1.7 km·h^−1^; *p* = 0.847; ES = 0.00, trivial), % maximal aerobic speed (97.2 ± 3.0 versus 97.0 ± 3.6 km·h^−1^; *p* = 0.823; ES = 0.00, trivial). 

### 3.3. Surface Electromyography

Muscular activity did not any reach statistical significance (Table 1). However, according to effect sizes the electromyographic activity was greater in the calf musculature when not using GCSs while running at submaximal effort, while descriptive changes were observed in effect size (ES). EA was lower in the leg during submaximal efforts (GAM and SOL, ES = 0.10, trivial) compared to in the thigh (VAL, VAM, BF, and RF, ES = 0.24, small) with GCS use versus CNT; leg (ES = 0.25, small) and leg (ES = 0.25, small). 

During running maximal effort (i.e., t1km), EA was higher in the leg (GAM and SOL, ES = 0.24, small) compared to in the thigh (VAL, VAM, BF, and RF, ES = 0.11, trivial) with GCS use versus CNT: thigh (ES = 0.17, trivial) and leg (ES = 0.18, trivial). (Table 1)

## 4. Discussion

The aim of this study was to evaluate changes in electromyographic activity with the use of the use of gradual compression stockings (GCSs) on endurance athletes’ performance, with the use of electromyography techniques. At the metabolic domain, our results are consistent with previous studies, where GCS use did not affect blood lactate concentration measurement [6,7,8,13]. Thus, performance variables during t1km reported no benefits from GCS use with regards to the time required to run the specified distance, and our data are in agreement with studies previously published by Ali [5,7], in which no differences were found in a 10 km test. Therefore, in our study there were no significant changes in the perceptual variables with GCS use, and small effect sizes were found for GCS use during submaximal efforts (*t* > VT2; ES = 0.33) and maximal efforts (t1km; ES = 0.39). This is consistent with previous studies, where lower muscle pain [5,18], greater comfort [6,7], and a lower subjective perception of effort [13,18,28] were observed. 

With regard to electromyography activity, it was evaluated by means of changes in amplitude (electromyography amplitude), since it has been described as a variable that provides knowledge on the degree of muscle fatigue [29]. No significant changes were found between treatments when measuring EA by surface electromyography, null and small effect sizes were observed (ES = 0.11–0.25), so that muscle activation changed according to whether CGSs were used or not. Thus, in submaximal efforts (*t* > VT2), the activation of the calf muscles (GAM, SOL) was observed to be less than that of the thigh muscles (VAL, VAM, RF, BF). This would have advantages for performance, as fatigue in the leg muscles frequently tends to limit performance more than that of the thigh muscles, because the gastrocnemius and soleus are the greatest contributors to propulsion and support during submaximal running [30]. According to our data, Lucas-Cuevas AG [31] reported a decrease in the muscular contribution of the GAM using GCSs in the rest situation and at the beginning of submaximal effort in the running race. However, one limitation of this study was that they did not analyze the possible changes in muscle recruitment between the thigh and muscular fatigue between GAM and SOL that we measured in our study.

During maximal efforts (t1km), muscle recruitment differs from that of submaximal efforts (*t* > VT2). The use of GCSs reduces electromyographic activity in the thigh and increases in the gastrocnemius muscles (sum GAM and SOL). If we analyze EA by muscle group in our study during the maximum effort of race (t1km), there was less recruitment using GCSs in both the BF and the RF than where there was no use of GCSs. This could be beneficial since in foot race efforts—as the speed increases, the RF and BF are the muscle groups that increase their muscular contribution the most [32,33]. 

During the present study, the study subjects realized a protocol for detecting electromyography activity performance, and effort perception variables during two different endurance tests. There are a few limitations that need to be addressed. First, it could be that the different tests used could not represent the endurance effort made by the athletes in a race. Thus, it could be necessary to gather more studies. Also, the individual data were variable, and the sample size was small and medium. Therefore, the lack of statistical significance could be due to a Type II error. Secondly, quantification of muscle activity from surface EMG signals is problematic when movement is involved and motion artifacts and other electromagnetic noises may influence the signal levels. We tried to minimize disturbances by the applied signal processing and filtering routines; still, artifacts may have small impacts on the derived maximum muscle activations. Thirdly, we could not use a multi-channel approach to provide access to a set of physiologically relevant variables on the global muscle level or on the level of single motor units, opening new fronts for the study of muscle fatigue; however, in the present study, we did not have this electromyographic analysis technology.

From our point of view, the various findings reported on previous studies with CGSs may be due to the different gradual compression socks used [5,6,7,8,12]. Other authors did not specify which type of GCSs they used in their studies; most likely they used stockings with a uniform degree of compression. 

All the studies conducted to date assessed the potential benefits of using compression means (GCS) during the efforts in the race on metabolic or cardiovascular variables. According to a study developed by Varela-Sanz et al. [13], there seems to be a tendency to run faster with a lower perception of effort. If there are no clear metabolic or cardiovascular benefits, the benefits can be found in a change in muscle recruitment between the thigh and leg muscle (i.e., triceps surae). Only a previous study developed by Lucas Cuevas et al. [31] analyzed muscle fatigue using GCSs by EMG techniques. However, Lucas Cuevas et al. [31] did not compare the possible recruitment changes between the muscles of the thigh; the study was realized with lower performance level athletes and lower intensities (75% maximal speed). The novelty of our study is the analysis of EMG recruitment on thigh muscle in high-level athletes at submaximal/maximal intensities. 

## 5. Conclusions

Endurance athletes perform much of their training and competitions at submaximal intensities Contrary to our initial hypothesis, no differences were reported on any of the variables analyzed on this study in EMG recruitment in well-trained athletes between the different conditions (GCSs versus CNT) at submaximal/maximal efforts. Future studies should be developed based on research findings to confirm our data or even explore other possibilities, such as the analysis of EMG recruitment during the recovery process, that are essential for performance in high-level athletes. 

## Figures and Tables

**Table 1:** EMG (electromyography) variables base on wearing or not wearing a graduate compression garment during the *t* > Vt2 and t1km.

	EMGrms (mV)												
	GCSs							CNT					
	*t* > VT2			t1km			*t* > VT2				t1km		
BF	0.320	±	0.181	0.314	±	0.196	0.323		±	0.104	0.304	±	0.304
RF	0.199	±	0.058	0.194	±	0.081	0.220		±	0.085	0.192	±	0.094
VAL	0.176	±	0.075	0.168	±	0.087	0.195		±	0.086	0.189	±	0.088
VAM	0.223	±	0.053	0.212	±	0.077	0.212		±	0.077	0.235	±	0.064
SOL	0.196	±	0.116	0.164	±	0.062	0.296		±	0.115	0.295	±	0.141
GAM	0.388	±	0.289	0.331	±	0.257	0.268		±	0.101	0.233	±	0.092

Abbreviations: vastus lateralis (VAL), vastus medialis (VAM), rectus femoris (RF), biceps femoris (BF), gastrocnemius (GAM), and soleus (SOL).
